# Global Heat Wave Hazard Considering Humidity Effects during the 21st Century

**DOI:** 10.3390/ijerph16091513

**Published:** 2019-04-29

**Authors:** Xi Chen, Ning Li, Jiawei Liu, Zhengtao Zhang, Yuan Liu

**Affiliations:** 1 Key Laboratory of Environmental Change and Natural Disaster of Ministry of Education, Faculty of Geographical Science, Beijing Normal University, Beijing 100875, China; summer_0409@foxmail.com (X.C.); ryanliu_xy@163.com (Y.L.); 2 Academy of Disaster Reduction and Emergency Management, Ministry of Emergency Management & Ministry of Education, Faculty of Geographical Science, Beijing Normal University, Beijing 100875, China; 3 Collaborative Innovation Center on Forecast and Evaluation of Meteorological Disasters (CIC-FEMD)/ Key Laboratory of Meteorological Disaster, Ministry of Education (KLME)/Joint International Research Laboratory of Climate and Environmental Change (ILCEC), Nanjing University of Information Science and Technology, Nanjing 210044, China; liu_jiawei@foxmail.com; 4 Institute of Geographic Sciences and Natural Resources Research, Chinese Academy of Sciences (CAS), Beijing 100101, China; zzt46@163.com

**Keywords:** heat waves, humidity, future scenarios, global

## Abstract

Humidity is a significant factor contributing to heat stress, but without enough consideration in studies of quantifying heat hazard or heat risk assessment. Here, the simplified wet-bulb globe temperature (WBGT) considering joint effects of temperature and humidity was utilized as a heat index and the number of annual total heat wave days (HWDs) was employed to quantify heat hazard. In order to evaluate the humidity effects on heat waves, we quantified the difference in the number of HWDs over global land based on air temperature and WBGT. Spatial and temporal changes in surface air temperature, relative humidity, WBGT, and the difference in HWDs were analyzed using multi-model simulations for the reference period (1986–2005) and different greenhouse gas emission scenarios. Our analysis suggests that annual mean WBGT has been increasing since 1986, which is consistent with the rising trend in surface air temperature despite a slight decrease in relative humidity. Additionally, changes in annual mean WBGT are smaller and more spatially uniform than those in annual mean air temperature as a cancelation effect between temperature and water vapor. Results show that there is an underestimation of around 40–140 days in the number of HWDs per year in most regions within 15° latitude of the equator (the humid and warm tropics) during 2076–2095 without considering humidity effects. However, the estimation of HWDs has limited distinction between using WBGT and temperature alone in arid or cold regions.

## 1. Introduction

The probability and intensity of extreme heat waves have been increasing over many parts of the world owing to climate change [1]. There is much more previous work on heat waves focusing on surface air temperature alone [2,3,4]. However, in addition to extreme high temperatures, surface relative humidity is also an important factor in defining heat waves as it is directly related to human body heat exchange either [5,6].

On the one hand, a resting human body generates about 100 W of metabolic heat (in addition to any absorbed solar heating) and cannot dissipate heat if the ambient temperature is higher than the optimum body core temperature (near 37 °C) owing to the second law of thermodynamics [7]. On the other hand, sweating, the main process by which the body transports away the environmental and metabolic heat loads, will become significantly less effective if the environmental relative humidity is high, resulting in heat accumulation in the body [8] and increases in both morbidity and mortality [5]. Thus, heat stress can occur in environment of temperatures lower than the optimum body core temperature accompanied by high relative humidity. As pointed out by Mora et al., with increasing relative humidity, the boundary at which temperature becomes deadly decreases, and some mortality events related to heat stress occurred at relatively low temperatures [9]. The above-mentioned facts are why a variety of heat stress indices like humidex [10], apparent temperature [11], and wet-bulb globe temperature [12] take both temperature and humidity into consideration. 

Wet-bulb globe temperature (WBGT) is the most common index including both temperature and humidity effects to quantify heat stress and has a long history of use. It was invented during the 1950s to control heat illness in training camps of the United States Army and Marine Corps [8] and was standardized by ISO 7243 for quantifying thermal comfort [13]. The heat stress index WBGT (°C) is a combination of the black globe temperature (measured inside a 15 cm diameter black globe), the natural wet bulb temperature (measured with a wetted thermometer exposed to the wind and heat radiation at the site), and the air temperature (measured with a “normal” thermometer shaded from direct heat radiation), to take into account the effect of heat absorption from the sun, and evaporative cooling, which is strongly related to air humidity. As ambient WBGT approaches the human body skin temperature, it becomes more difficult for the body to dissipate heat to an environment [14]. 

Recent studies have pointed to a growing concern on increasing heat stress considering humidity effects as well as extreme temperatures. Kang and Eltahir emphasized the important role of humidity, and pointed out that North China Plain is likely to experience deadly heat waves with wet-bulb temperature exceeding the threshold defining what Chinese farmers may tolerate while working outdoors [15]. By applying 35 °C as a threshold for human adaptability, Pal and Eltahir predicted that extremes of wet-bulb temperature in the region around the Arabian Gulf are likely to approach and exceed this threshold under business-as-usual emission scenarios [16]. Lin et al. determined trends of heat wave variation and stress threshold in three major cities of Taiwan based on WBGT, and suggested that the heat stress in all three cities will either exceed or approach the danger level (WBGT ≥ 31 °C) by the end of this century [17]. Russo et al. quantified humid heat wave hazards in the recent past and at different levels of global warming using the apparent temperature, showing that humidity can amplify the magnitude and apparent temperature peak of heat waves [11]. There have also been some studies assessing the adverse effects of heat stress on health and labor productivity [18,19]. 

Despite the need for effective and scientific information about heat stress considering humidity effects, the assessment and comparative analysis on a global scale has been limited so far. Therefore, this study aims to analyze the future changes in heat wave hazard over global land using multi-model simulations integrated under different greenhouse gas emission scenarios. Here, WBGT was utilized as a heat index and the number of annual total heat wave days (HWDs) was employed to quantify the heat hazard [20]. We focus on the spatial distribution and temporal variation of difference in heat hazard simulated by WBGT and only temperature independently in order to evaluate the effects of humidity on heat events.

## 2. Data and Methods

Daily data of mean surface air temperature and near-surface relative humidity from multi-model simulations (Table 1) for the Coupled Model Inter-comparison Project Phase 5 (CMIP5) were used in this study, which can be available from the Earth System Grid Federation (ESGF) (https://esgf-index1.ceda.ac.uk/search/cmip5-ceda/). All the data were interpolated to a common 0.5° grid cell size using a bilinear function. We used one run (rli1p1) for each model.

In this study, WBGT was used as a heat index following previous studies on long-term changes in heat stress [12,14,21] and the number of total heat wave days (HWDs) in a year was employed to quantify the heat hazard [20]. Equation (1) is used to evaluate the heat stress in the outdoors where solar radiation is present, and Equation (2) is used to evaluate the heat stress in the indoors or outdoors without solar radiation:

(1) $$\mathrm{WBGT}=0.7T_{nwb}+0.2T_{g}+0.1T_{a}$$

(2) $$\mathrm{WBGT}=0.7T_{nwb}+0.3T_{g}$$

where $T_{nwb}$ is natural wet bulb temperature (°C), $T_{g}$ is black globe temperature (°C), and $T_{a}$ is air temperature (°C). Owing to the difficulty to obtain black globe temperature [22], so we used the ‘simplified WBGT’ (hereafter denoted simply as ‘*W*’, as in Equation (3) below), which was developed by the American College of Sports Medicine [23]. *W* depends only on air temperature and relative humidity, and represents heat stress for average daytime conditions outdoors:

(3) $$W=0.567T_{a}+0.393e+3.94$$

(4) $$e=\left(\mathrm{RH}/100\right)\times 6.105\exp\left(\frac{17.27T_{a}}{237.7+T_{a}}\right)$$

where e is water vapour pressure (hPa) and RH is relative humidity (%). Here, water vapor pressure was calculated based on daily air temperature and relative humidity, and ignores the impact of wind and radiation on thermal stress. However, wind and radiation are unlikely to have a significant systematic effect globally on the trend of heat stress [12]. 

We first calculated daily *W* during the reference period (1986–2005) and the future period (2006–2095) using daily simulations from 19 CMIP5 GCMs under three Representative Concentration Pathways 2.6, 4.5 and 8.5 (RCP2.6, RCP4.5 and RCP8.5, respectively) on each grid. In climatology, the occurrence of at least three consecutive hot days has been defined as a heat wave [24,25]. The number of annual total HWDs, employed to quantify heat wave hazard, is defined as the total days of heat waves in a year. In order to evaluate the influence of humidity on heat waves, we calculated the number of annual total HWDs based on air temperature (hereafter denoted simply as ‘THWDs’) and *W* (hereafter denoted simply as ‘WHWDs’) considering both temperature and humidity effects, respectively. In this study, a heat wave is considered as daily air temperature/*W* exceeding a given region’s threshold for at least three consecutive days. We calculated the local 95th percentile value of daily air temperature/W per year over the reference period and the threshold is defined as the average of these 95th percentile values. 

## 3. Results

### 3.1. Fidelity of 19 CMIP5 GCMs Used

**Figure 1:**
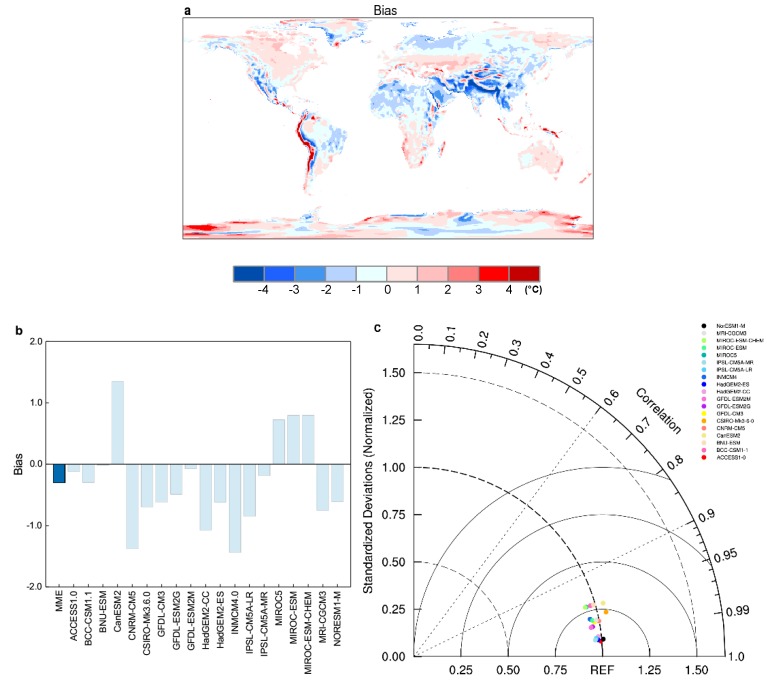
Spatial distribution of climatology of annual mean WBGT during the reference period (1986–2005) from (**a**) CMIP5 MME bias. (**b**) Area mean bias and (**c**) Taylor diagram for WBGT for CMIP5 individual models in comparison with ERA-Interim reanalysis.

Before examining future changes of heat hazard, we have evaluated CMIP5 models in terms of WBGT climatology in comparison with the ERA-Interim reanalysis. Bias and the Taylor diagram analysis [26] were used to evaluate model skills (Figure 1). Results show that the annual mean WBGT over global land is well simulated by CMIP5 models (Figure 1 and Supplementary Figure S1), with some differences in areas having large terrain undulations. MME bias is −0.3 °C, with some differences across models. The Taylor diagram used in this study relates two statistical indicators of model fidelity: Spatial standard deviations and correlations, indicating the relative amplitude of the simulated and the reference variations, and the degree of similarity of variation between the two, respectively. Both the standard deviation and correlation of the reference data set are equal to 1. Any deviations from 1 for either of them represent a mismatch of modeled variables from the reference data. Figure 1c shows that the spatial correlation coefficients of all models are greater than 0.95, and the standard deviations of spatial variability are also very similar to the reanalysis. 

### 3.2. Future Changes in Air Temperature, Relative Humidity and W

**Figure 2:**
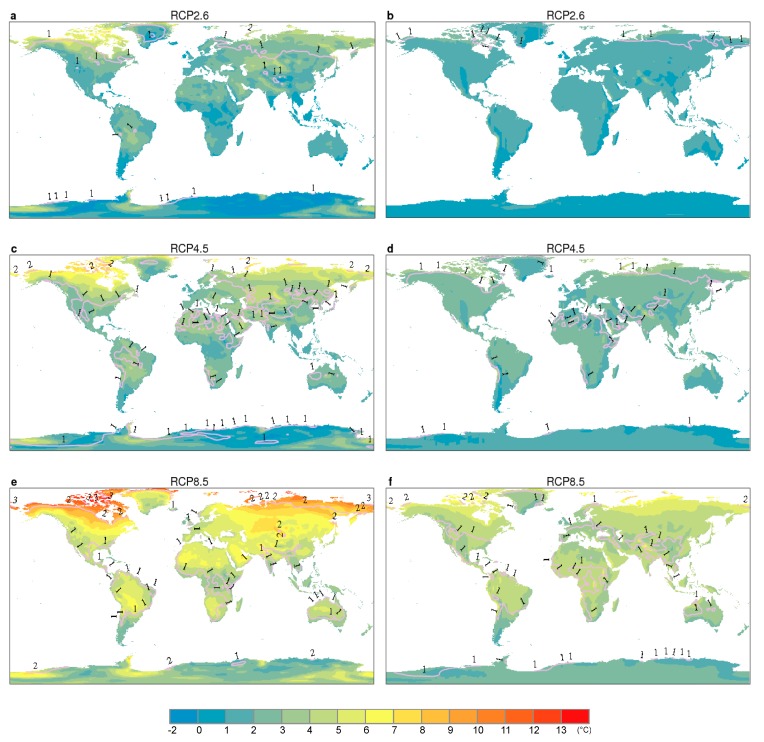

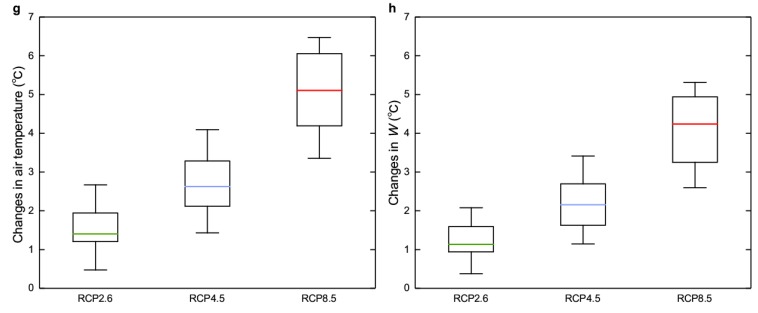
Changes in annual mean air temperature (shading) during 2076–2095 relative to 1986–2005 and multi-model standard deviation (contour) under (**a**) RCP2.6, (**c**) RCP4.5, and (**e**) RCP8.5. (**g**) shows the corresponding range of projected spatially averaged increases in annual mean temperature over global land based on the results of CMIP5 GCMs used. Panels (**b**,**d**,**f**,**h**): same as (**a**,**c**,**e**,**g**) except for annual mean *W*.

Annual mean *W* over global land has been increasing since 1986 (Supplementary Figure S2a), which is consistent with the rising trend in surface air temperature (Supplementary Figure S2b) despite a slight decrease in relative humidity (Supplementary Figure S2c). In addition, more decreases in annual mean relative humidity was detected under higher emission scenarios (Supplementary Figure S2c). Figure 2 shows the spatial distributions of changes in mean air temperature and *W*, and the corresponding range of projected spatially averaged changes. We found that by 2076–2095, both annual mean air temperature and *W* rising almost around the global land and the largest increases in both of them occur at high northern latitudes under all emission scenarios (Figure 2a–f). In contrast to the global warming, relative humidity exerts slightly decreases over most land regions (Supplementary Figure S4a–c) in accordance with the Clausius–Clapeyron relationship, which supports previous studies finding a decrease in surface relative humidity over land regions [27,28]. 

While GCMs show larger uncertainties for the humidity simulation compared to air temperature (Figure 2a,c,e and Supplementary Figure S3a–c), multi-model standard deviation of *W* which includes both temperature and humidity is smaller than the uncertainty in each variable if analyzed independently (Figure 2 and Supplementary Figure S3a–c), according with the research result of Fischer and Knutti [29]. Models also suggest that the changes in annual mean *W* under the three RCP scenarios are overall smaller, more spatially uniform, and thus have less inter-model variations than those in annual mean air temperature (Figure 2 and Supplementary Figure S4). This is because GCMs that simulate more warming also tend to show larger decreases in relative humidity (Supplementary Figures S2c and S3d) [29], generating a cancelation effect between air temperature and water vapor. The result is consistent with previous studies that found the spread of changes in air temperature was substantially larger than that in WBGT from both observations and models [12,14,30].

### 3.3. Differences between WHWDs and THWDs

**Figure 3:**
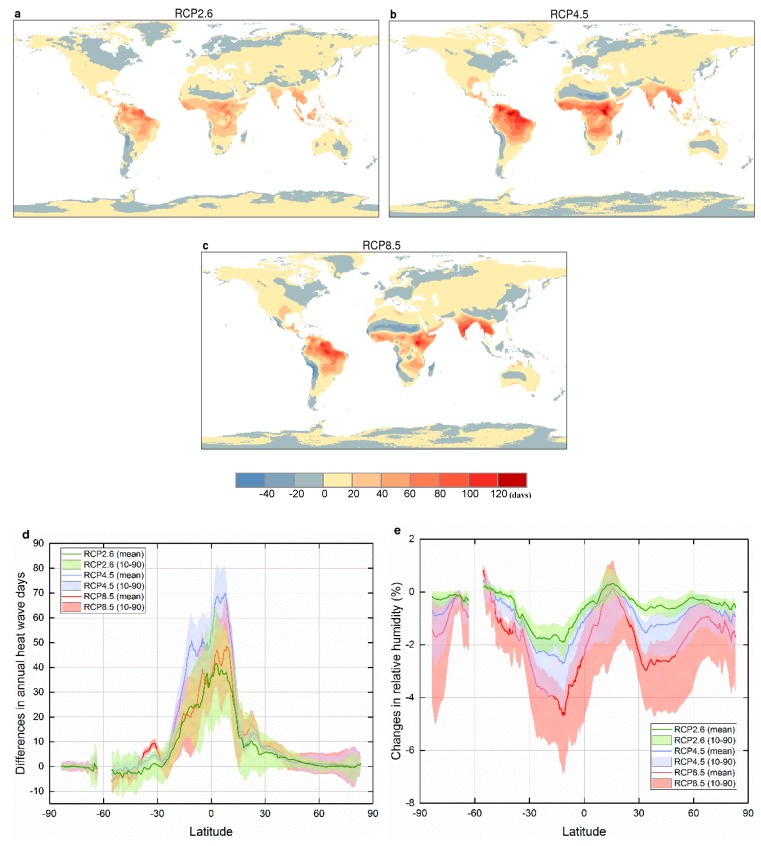
The spatial distributions of difference in annual total WHWDs and THWDs during 2076–2095 under (**a**) RCP2.6, (**b**) RCP4.5, and (**c**) RCP8.5. (**d**) shows the variation with latitude of the difference over land during 2076–2095 under all three scenarios. (**e**) shows the variation with latitude of the change in relative humidity during 2076–2095, relative to mean values between 1986 and 2005. Bold lines are the multi-model averages, shaded areas are the 10–90% expected ranges of the CMIP5 GCMs used.

In order to evaluate the influence of humidity on heat waves, we subtracted annual total THWDs from annual total WHWDs on each grid cell and illustrated the spatial distributions of MME result during 2076–2095 under all three scenarios (Figure 3a–c). We can find that annual WHWDs are greater than THWDs over the most global land, especially at the equator and its adjacent regions. By 2076–2095, most regions near the equator would experience about 40–140 WHWDs more than THWDs in a year under RCP4.5. In the mid-latitudes, the discrepant days per year would be somewhat lower at 0–20 or negative under all three scenarios. Figure 3c displays the variation with latitude of the difference in the number of annual total WHWDs and THWDs during 2076–2095, excluding water grid cells. Results show that the zonally averaged difference between WHWDs and THWDs exerts a decreasing trend from the equator to the middle and high latitudes. The difference in the number of HWDs is very small (almost to zero) in south of 30° S and North of 60° N, and in contrast, the number is approximately between 0 and 70 in the regions within 30° latitude of the equator. 

It is worth noting that the number of days under RCP4.5 in the regions within 30° latitude of the equator is more than that under RCP8.5 on the whole, and the variation with latitude of the number under RCP2.6 is similar to that under RCP8.5. Figure 3e displays the zonally averaged change in annual mean relative humidity during 2076–2095, relative to mean values between 1986 and 2005. We can find that except for a slight increase within 10° N and 20° N, relative humidity would decrease at most latitudes and there would be a larger decrease (that is, a lower relative humidity) under RCP8.5, which could result in less difference in the number of WHWDs and THWDs in the humid tropics than that under RCP4.5. Analogously, the less decrease in relative humidity (that is, a higher relative humidity) in the humid tropics under RCP2.6 contributes to a similar variation to that under RCP8.5. 

**Figure 4:**
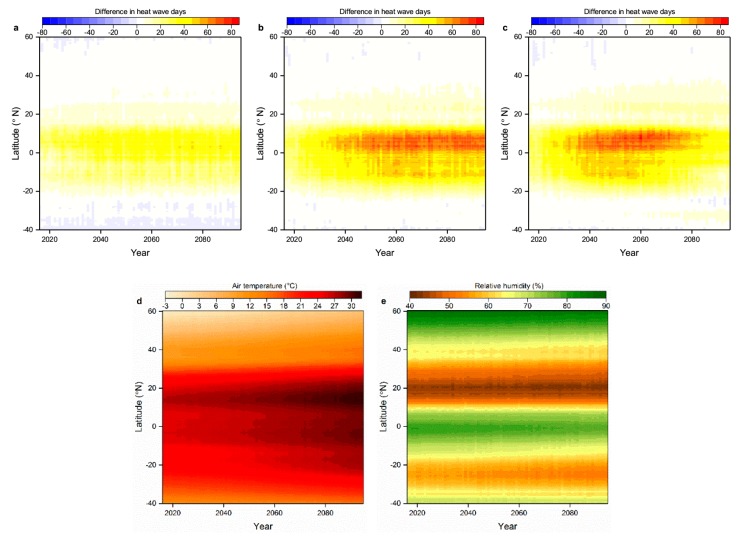
Temporal changes with latitude in the difference in the number of annual WHWDs and THWDs over land under (**a**) RCP2.6, (**b**) RCP4.5, and (**c**) RCP8.5. (**d**) Mean air temperature and (**e**) relative humidity under RCP8.5 were also simulated. Results are based on the multi-model averages during 2016–2095.

We also evaluated temporal variation with latitude of the difference in annual total WHWDs and THWDs under all three scenarios (Figure 4a–c). Between 2016 and 2095, regions within around 10° latitude of the equator are with large difference (about 40–80 days) in a year under all scenarios, which have high relative humidity and consistently warm temperatures (Figure 4d–e). In spite of a slight increase in relative humidity near 20° N (Supplementary Figure S5c,f,i), the number of discrepant days is very small due to very low humidity inherently there (Figure 4e). Analogously, although northern high latitudes (near 60° N) have high relative humidity (Figure 4e) and will experience more warming than tropical areas (Supplementary Figure S5b,e,h) [31], owing to having relatively low air temperature, the difference in the number of WHWDs and THWDs is also limited. The above consequences suggest that the estimation of heat wave days based only on temperature may result to an underestimation in humid and warm regions but may have limited effects in arid or cold regions.

By 2016–2095, the zonally averaged difference in the number of WHWDs and THWDs under RCP2.6 is smaller than the other two scenarios on the whole as less warming (Supplementary Figure S5b,e,h) contributes to both less WHWDs and THWDs. The largest difference (about 60–80 days) would begin from around 2030 to 2075 under RCP 8.5, which would continue till 2095 under RCP4.5. This is in accord with Figure 3d that suggests the larger zonally averaged number of discrepant days under RCP4.5 than RCP8.5 during 2076–2095. 

## 4. Discussion

On a global scale, greenhouse-gas-induced warming generates an increase in the near-surface absolute humidity of the air according to the Clausius–Clapeyron relationship, and thus relative humidity remains roughly constant or slightly decreases over land [32]. One possible cause is that the reduction from a limited moisture supply over land because of less and slower ocean warming relative to land warming over the period [14]. However, local relative humidity may change due to general circulation changes [32,33]. For example, as a heat stress hot spot, coastal Middle East may be influenced by the advection of hot air masses from desert areas and the water vapor advection from warm water bodies [16]; in regions such as the Eastern US and China, the formation of a humid heat wave is typically due to hot and humid air advected from the Gulf of Mexico or from tropical regions, respectively [34]. These processes occur at too small scales to be captured by GCMs, which may potentially add a conservative bias to our results. 

Soil moisture can affect the partitioning of net radiation in latent and sensible heat fluxes, and the lack of it will reduce evaporative cooling and thereby amplify extreme high temperatures. Some modelling studies have postulated a possible impact of soil-moisture deficit and drought on hot extremes [35,36]. According to Mora et al., tropical areas have prominently higher soil moisture compared to mid-latitudes, and increasing sensible heat flux amplifies extreme high temperatures at mid-latitudes owing to the lack of soil moisture there [9]. The implications for extreme WBGT and heat waves considering soil moisture poorly simulated by GCMs need to be explored in the future. 

Our initial exploration of differences in the number of heat wave days simulated by WBGT and only temperature independently under the three RCPs has some limitations. A challenge faced by heat wave studies is that no standard definition exists in terms of temperature threshold, metric, and lasting days. We only use the 95th percentile and at least three consecutive days to define a heat wave, and don’t conduct a sensitivity analysis through using a series of percentiles and consecutive days, though the increase in heat wave metrics under global warming is essentially insensitive to the percentile threshold chosen [37]. Additionally, thermal comfort is a complicated matter and depends on various variables, such as age, health, gender, type and amount of clothing, acclimatization, and also individual tolerance [12]. We only evaluate the climatic conditions considering both temperature and humidity effects without taking into the effect of adaptation or vulnerability. Although WBGT has been assessed to have some applicability worldwide, some other heat indexes considering both temperature and humidity effects such as humidex [10], apparent temperature [11], wet-bulb globe temperature [12] have not been used in this study. We will carry out research about whether utilizing different heat indices will change heat wave characteristics or not in the near future. 

## 5. Conclusions

Our study underscores the consideration of humidity effects in defining heat waves and quantifying heat wave hazard, especially in the humid and warm tropics. We find that annual mean *W* has been increasing over global land since 1986, which is consistent with the increase in surface air temperature despite a decrease in average relative humidity. Although GCMs show larger inter-model variations for the humidity simulation compared to air temperature, multi-model standard deviation of *W* is smaller than the uncertainty in each variable if analyzed independently. Additionally, the cancelation effect between air temperature and water vapor results in the changes in annual mean *W* smaller and more spatially uniform than those in annual mean air temperature. Results show that there will be an underestimation of around 40–140 days in the number of heat wave days per year in most regions within 15° latitude of the equator (humid and warm tropics) during 2076–2095 without considering humidity effects. However, the estimation of heat wave days based only on temperature may have limited effects in very arid or cold regions. Our paper emphasizes the importance of humidity effects other than air temperature in defining heat waves in humid and warm regions, and provides scientific information for further humid heat risk assessment.

## Figures and Tables

**Table 1:** Overview of the GCMs used in this study.

Model	Center and Country	Historical	RCP2.6	RCP4.5	RCP8.5
ACCESS1.0	CSIRO-BOM, Australia	**√**	**-**	**√**	**√**
BCC-CSM1.1	BCC, China	**√**	**√**	**√**	**√**
BNU-ESM	BNU, China	**√**	**√**	**√**	**√**
CanESM2	CCCma, Canada	**√**	**√**	**√**	**√**
CNRM-CM5	CNRM-CERFACS, France	**√**	**√**	**√**	**√**
CSIRO-Mk3.6.0	CSIRO-QCCCE, Australia	**√**	**√**	**√**	**√**
GFDL-CM3	NOAA-GFDL, USA	**√**	**√**	**√**	**√**
GFDL-ESM2G	NOAA-GFDL, USA	**√**	**√**	**√**	**√**
GFDL-ESM2M	NOAA-GFDL, USA	**√**	**√**	**√**	**√**
HadGEM2-CC	MOHC, UK	**√**	**-**	**√**	**√**
HadGEM2-ES	MOHC, UK	**√**	**√**	**√**	**√**
INMCM4.0	INM, Russia	**√**	**-**	**√**	**√**
IPSL-CM5A-LR	IPSL, France	**√**	**√**	**√**	**√**
IPSL-CM5A-MR	IPSL, France	**√**	**√**	**√**	**√**
MIROC-ESM	MIROC, Japan	**√**	**√**	**√**	**√**
MIROC-ESM-CHEM	MIROC, Japan	**√**	**√**	**√**	**√**
MIROC5	MIROC, Japan	**√**	**√**	**√**	**√**
MRI-CGCM3	MRI, Japan	**√**	**√**	**√**	**√**
NorESM1-M	NCC, NMI, Norway	**√**	**√**	**√**	**√**

## Supplementary Materials

The following are available online at https://www.mdpi.com/1660-4601/16/9/1513/s1, Figure S1: Spatial distributions of climatology of annual mean daily mean WBGT during 1986–2005 from (a) MME of CMI5 historical runs and (b) ERA-Interim reanalysis, Figure S2: Time series of (a) annual mean W, (b) air temperature and (c) relative humidity over global land for the historical experiment and under all three scenarios. Bold lines are the multi-model averages, shaded areas are the 10–90% expected ranges of the CMIP5 GCMs used in this study. All the results were calculated as the area-weighted global averages, Figure S3: Changes in annual mean relative humidity during 2076-2095 relative to 1986–2005 and multi-model standard deviation (contour) under (a) RCP2.6, (b) RCP4.5 and (c) RCP8.5, and (d) the corresponding spatially averaged over land based on the results of CMIP5 GCMs used, Figure S4: Spatial distributions of the difference between Figure 2a,c,e and Figure 2b,d,f, respectively, namely, the difference between changes in annual mean air temperature and changes in annual mean W under (a) RCP2.6, (b) RCP4.5 and (c) RCP8.5, Figure S5: Temporal changes with latitude in the difference of annual total WHWDs and THWDs (left), air temperature (middle) and relative humidity (right) under (a–c) RCP2.6, (d–f) RCP4.5 and (g–i) RCP8.5, relative to mean values between 1986 and 2005. Results are based on the multi-model averages during 2016–2095.
