# Supplementary material for: Global Heat Wave Hazard Considering Humidity Effects during the 21st Century
Source: Int J Environ Res Public Health. 2019 Apr 29;16(9):1513. doi: 10.3390/ijerph16091513 (PMC6539408; doi:10.3390/ijerph16091513)
Supplement: Supplementary file 1 [file ijerph-16-01513-s001.pdf]

International Journal of
Environmental Research and Public Health

Supplementary Information for

Global heat wave hazard considering humidity effects during the 21st century

Xi Chen[1,2], Ning Li[1,2], Jiawei Liu[3], Zhengtao Zhang[4], Yuan Liu[1,2]

1 Key Laboratory of Environmental Change and Natural Disaster of Ministry of Education, Faculty of Geographical Science, Beijing Normal University, Beijing 100875, China

2 Academy of Disaster Reduction and Emergency Management, Ministry of Emergency Management & Ministry of Education, Faculty of Geographical Science, Beijing Normal University, Beijing 100875, China

3 Collaborative Innovation Center on Forecast and Evaluation of Meteorological Disasters (CIC-FEMD)/ Key Laboratory of Meteorological Disaster, Ministry of Education (KLME)/ Joint International Research Laboratory of Climate and Environmental Change (ILCEC), Nanjing University of Information Science and Technology, Nanjing 210044, China

4 Institute of Geographic Sciences and Natural Resources Research, Chinese Academy of Sciences (CAS), Beijing 100101, China

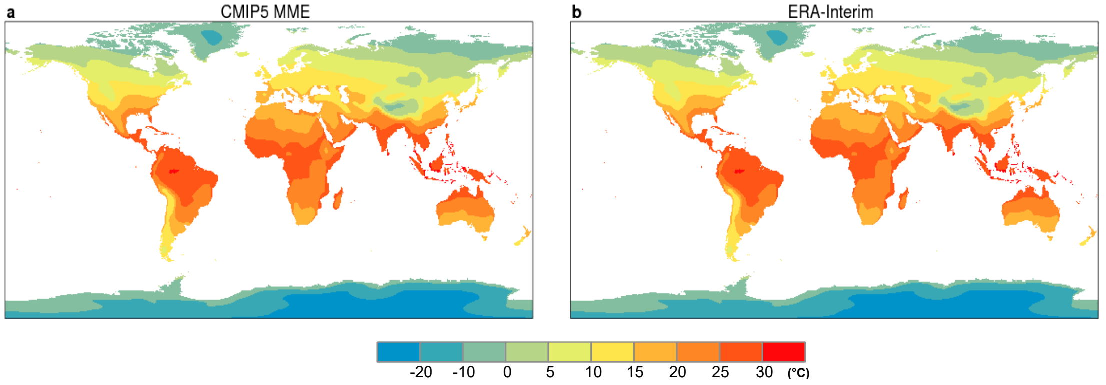

Figure S1. Spatial distributions of climatology of annual mean daily mean WBGT during 1986–2005 from (**a**) MME of CMI5 historical runs and (**b**) ERA-Interim reanalysis.

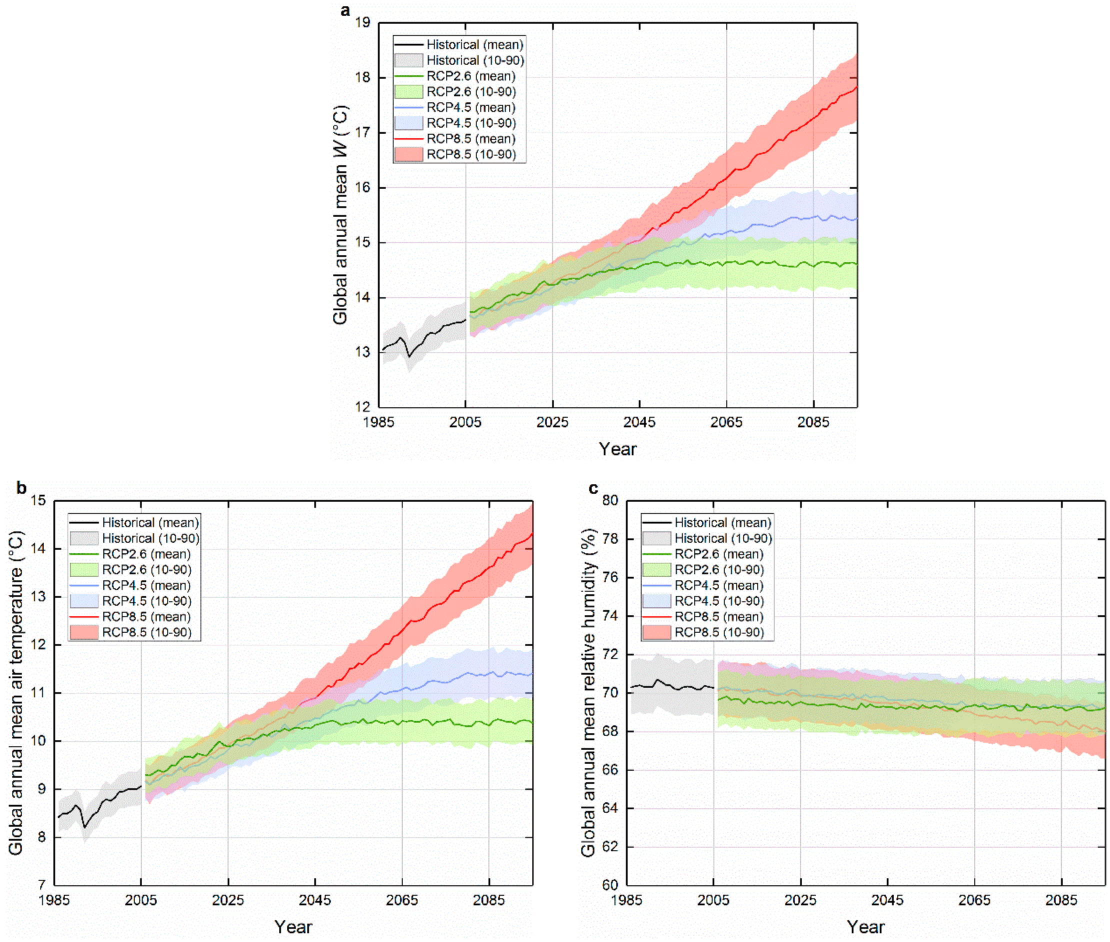

Figure S2. Time series of (**a**) annual mean *W*, (**b**) air temperature and (**c**) relative humidity over global land for the historical experiment and under all three scenarios. Bold lines are the multi-model averages, shaded areas are the 10–90% expected ranges of the CMIP5 GCMs used in this study. All the results were calculated as the area-weighted global averages.

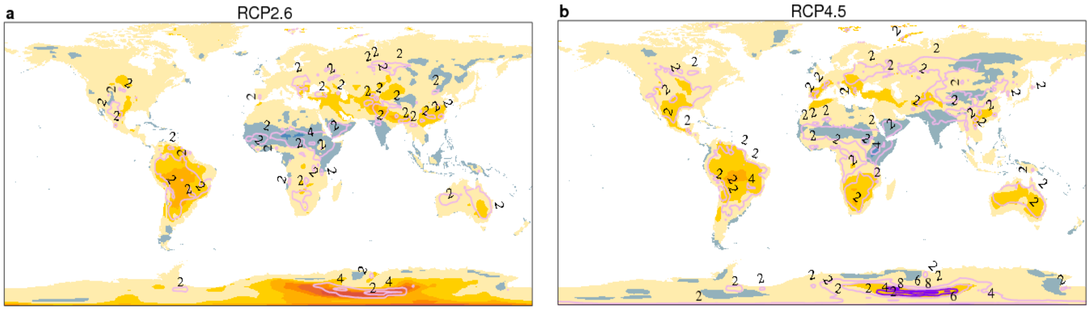

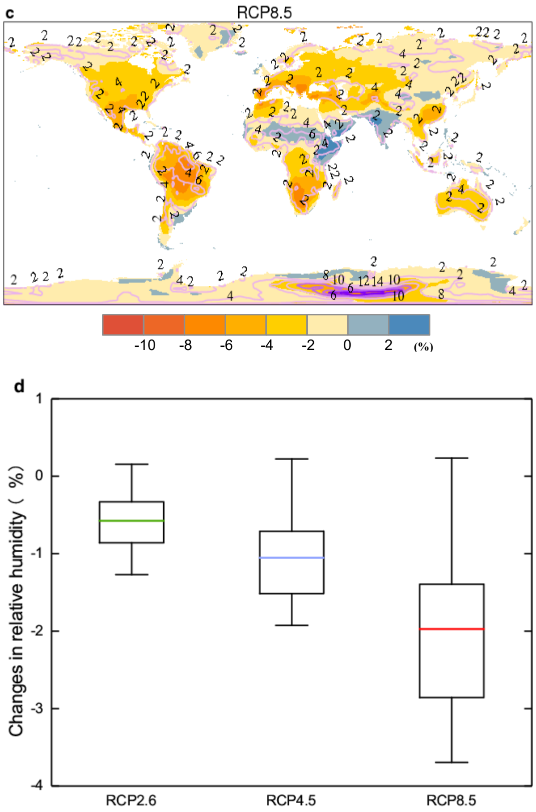

Figure S3. Changes in annual mean relative humidity during 2076-2095 relative to 1986–2005 and multi-model standard deviation (contour) under (**a**) RCP2.6, (**b**) RCP4.5 and (**c**) RCP8.5, and (**d**) the corresponding spatially averaged over land based on the results of CMIP5 GCMs used.

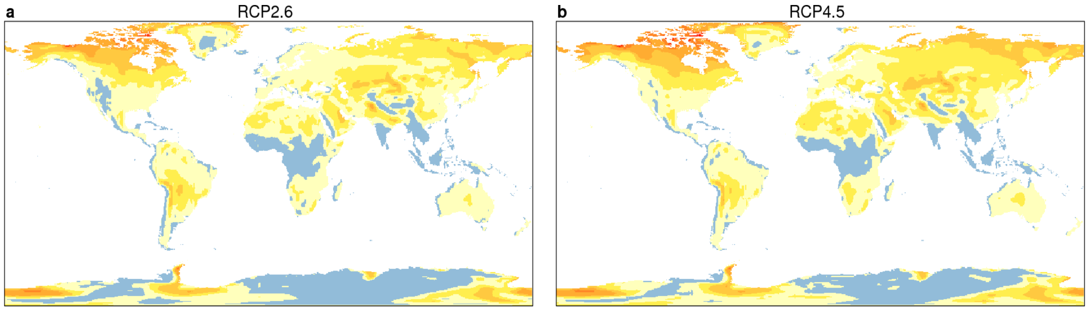

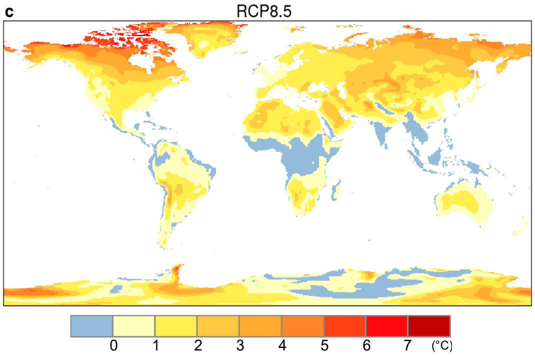

Figure S4. Spatial distributions of the difference between Figure 2a,c,e and Figure 2b,d,f, respectively, namely, the difference between changes in annual mean air temperature and changes in annual mean *W* under (**a**) RCP2.6, (**b**) RCP4.5 and (**c**) RCP8.5.

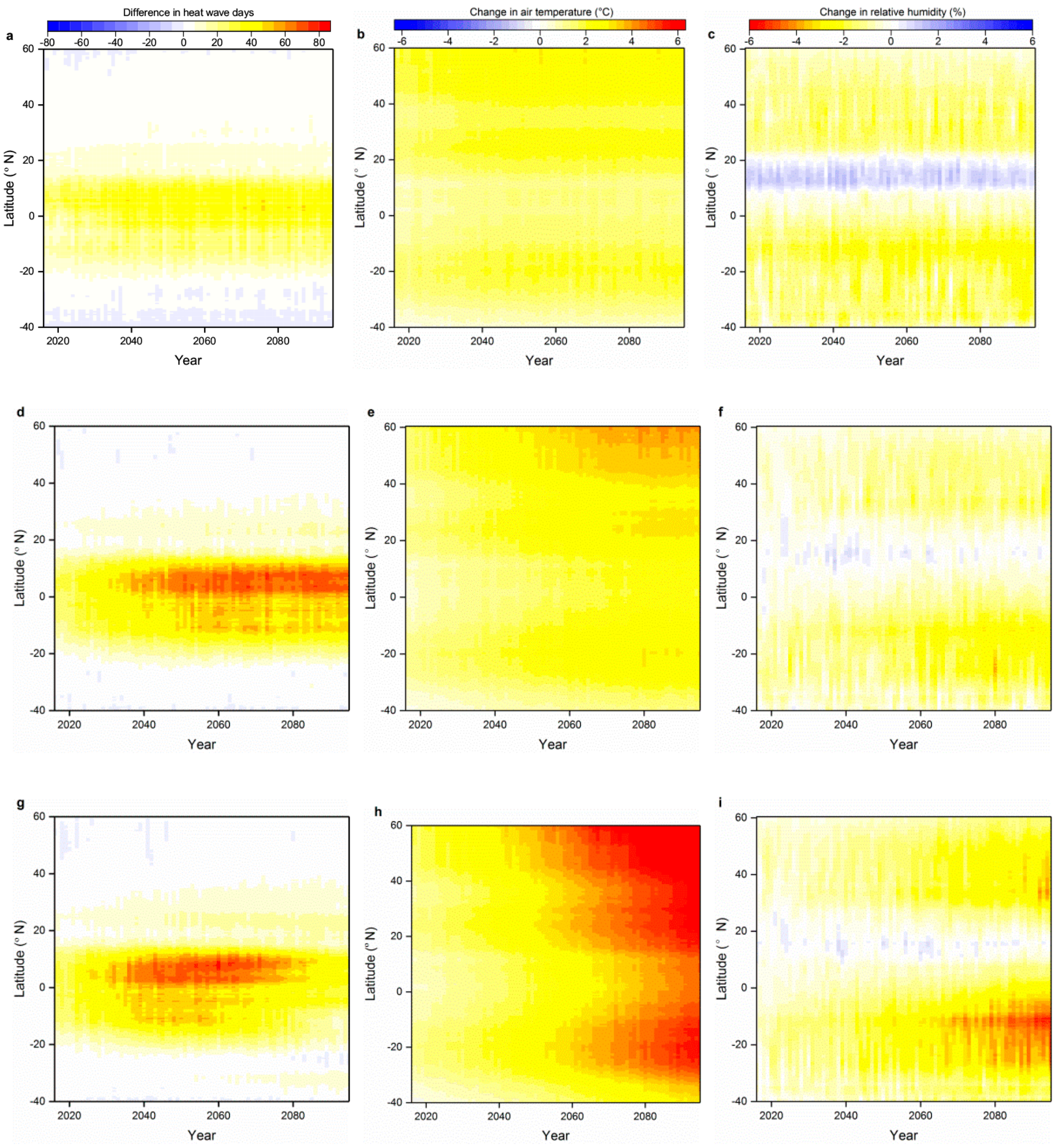

Figure S5. Temporal changes with latitude in the difference of annual total WHWDs and THWDs (left), air temperature (middle) and relative humidity (right) under (**a**–**c**) RCP2.6, (**d**–**f**) RCP4.5 and (**g**–**i**) RCP8.5, relative to mean values between 1986 and 2005. Results are based on the multi-model averages during 2016–2095.
